# Isolation and Characterization of the First Microsatellite Markers for the Endangered Relict Mussel *Hypanis colorata* (Mollusca: Bivalvia: Cardiidae)

**DOI:** 10.3390/ijms12010456

**Published:** 2011-01-17

**Authors:** Oana Paula Popa, Elena Iulia Iorgu, Ana Maria Krapal, Beatrice Simona Kelemen, Dumitru Murariu, Luis Ovidiu Popa

**Affiliations:** 1 “Grigore Antipa” National Museum of Natural History, Molecular Biology Department, Kiseleff Street, No 1, Bucharest 011341, Romania; E-Mails: elenap@antipa.ro (E.I.I.); ana.krapal@antipa.ro (A.M.K.); dmurariu@antipa.ro (D.M.); popaluis@antipa.ro (L.O.P.); 2 Department of Biochemistry and Molecular Biology, University of Bucharest, 91-95 Spl. Independentei, Bucharest 050095, Romania; 3 “Babes-Bolyai” University, Molecular Biology Center, 42 Treboniu Laurian Street, 400271 Cluj-Napoca, Romania; E-Mail: bea_kelemen@yahoo.com

**Keywords:** Monodacna, Ponto-Caspian, invasive species, population genetics, repetitive elements, genetic diversity

## Abstract

*Hypanis colorata* (Eichwald, 1829) (Cardiidae: Lymnocardiinae) is a bivalve relict species with a Ponto-Caspian distribution and is under strict protection in Romania, according to national regulations. While the species is depressed in the western Black Sea lagoons from Romania and Ukraine, it is also a successful invader in the middle Dniepr and Volga regions. Establishing a conservation strategy for this species or studying its invasion process requires knowledge about the genetic structure of the species populations. We have isolated and characterized nine polymorphic microsatellite markers in *H. colorata*. The number of alleles per locus ranged from 4 to 28 and the observed heterozygosity ranged from 0.613 to 1.000. The microsatellites developed in the present study are highly polymorphic and they should be useful for the assessment of genetic variation within this species.

## 1. Introduction

*Hypanis colorata* (Eichwald, 1829) (Cardiidae: Lymnocardiinae) is a bivalve relict species with a Ponto-Caspian distribution. The subfamily Lymnocardiinae includes several fossil genera and two extant genera, *Hypanis* and *Didacna*. In Romania, *Hypanis colorata* is a species under strict protection, according to national regulations. In the past the species had a wider distribution in the western Black Sea basin, but today the species is only present in several Black Sea lagoons in Romania and in Ukraine [1,2]. The species is also a successful invader in the middle Dniepr and Volga regions [3,4]. The literature regarding *H. colorata* is scarce and no data are available about the genetic diversity and population structure of the species.

Establishing biological conservation strategies requires knowledge of species populations genetic structure in order to identify evolutionary significant units (ESU), which are defined as a population or group of populations that deserve separate management or priority for conservation because of their high distinctiveness, both genetic and ecological [5]. In the case of invasive species, the study of their genetic diversity in the invaded area in comparison to the native area could help to infer important aspects of the invasion process, like the route(s) of invasion, the time of invasion, *etc*. Microsatellite markers are useful tools to investigate the genetic diversity in wild populations because they are highly polymorphic and codominant [6–8].

In this paper we describe, for the first time, nine polymorphic microsatellite loci in the species *Hypanis colorata.*

## 2. Results and Discussion

After screening the microsatellite enriched genomic library, we selected a number of 21 positive clones (that generated two bands on an agarose gel) which were further sequenced using the LICOR 4300L Genetic Analyzer. All of the sequenced clones contained microsatellite motifs with at least four uninterrupted repeats, but only 15 of them were found suitable for primer design after discarding duplicates, hybrid clones and those clones with too short flanking regions of the microsatellite array, unsuitable for primer design. Nine out of 15 primer pairs gave consistent amplification and were subsequently used for polymorphism screening (Table 1).

We tested the degree of polymorphism of the isolated loci in 32 individuals of *Hypanis colorata* collected from the Razelm-Sinoe Lake complex. The number of alleles per locus ranged from 4 to 28 and the observed heterozygosity ranged from 0.613 to 1.000 (Table 2). After applying the Bonferroni correction, one locus (Hypo13) showed a significant deviation from the Hardy-Weinberg equilibrium (HWE) (p < 0.05), while linkage disequilibrium was found between four pairs of loci out of 81 compared pairs (Hypo13 *vs*. Hypo10, Hypo13 *vs*. Hypo11, Hypo13 *vs*. Hypo2 and Hypo9 *vs*. Hypo5). All these instances of linkage disequilibrium were no longer statistically supported after Bonferroni correction. The results of the Micro-Checker testing showed that two of the loci (Hypo12 and Hypo5) exhibited an excess of homozygotes, most likely due to the presence of null alleles. This is a common reported phenomenon in bivalve species [9,10].

The microsatellites developed in the present study are highly polymorphic and they should be useful for further studies of conservation genetics, and may thus improve our understanding of the life history of these relict species. They can also be used to study how the genetic diversity of the invasive populations of *H. colorata* changes through space and time in comparison to native populations, which could lead to the identification of general features of the invasive process of this species.

Table 1 shows the primer sequences and characteristics of the nine microsatellite loci successfully amplified in *H. colorata*. Table 2 shows the variation across the nine microsatellite loci in *H. colorata*.

## 3. Experimental Section

The microsatellite loci were developed using a modified enrichment protocol [11,12]. Specimens of *H. colorata* were collected in 2009 from the Razelm Sinoe Lagoon and tissue samples were preserved in 95% ethanol. The genomic DNA was extracted from the muscular tissue of 5 individuals, using the NucleoBond^®^ AXG 100 Kit (Macherey-Nagel GmbH & Co. KG, Düren, Germany), according to the producer’s specifications. The different DNA extractions were pooled and then 9 μg of DNA was digested with the restriction enzyme Sau3AI (Jenna Bioscience GmbH, Jena, Germany) for two hours at 37 °C.

The isolated DNA fragments were ligated to double strand adaptors which were constructed from two different oligonucleotides mixed at a concentration of 25 μM each, denaturated at 80 °C for 5 minutes, then cooled down to room temperature for 1 hour [11]. The DNA sequence of the oligos is 5′-gatcgtcgacggtaccgaattct-3′ (OligoA) and 5′-gtcaagaattcggtaccgtcgac-3′ (OligoB). Adaptor-ligated DNA was run in a 2% agarose gel and fragments from 400 bp to 800 bp in size were excised and purified using the GeneJET™ Gel Extraction Kit (Fermentas UAB, Vilnius, Lithuania). The ligated fragments were submitted to a short (10 cycles) polymerase chain reaction (PCR) using OligoA as a primer, in order to increase the number of DNA fragments with ligated adaptors at both ends [12]. This PCR reaction was performed in 25 μL of a solution containing 10 mM Tris-HCl (pH 8.8 at 25 °C), 50 mM KCl, 0.08% (v/v) Nonidet P40, 2.5 mM MgCl_2_, each dNTP at 0.1 mM, OligoA primer at 10 μM, 1 unit of Taq DNA polymerase (Fermentas UAB, Vilnius, Lithuania) and approximately 20 ng of DNA template. The temperature profile of the polymerase chain reaction consisted of an initial denaturation at 94 °C for 30 s, followed by 10 cycles at 95 °C for 50s, 65 °C for 1min, 72 °C for 2 min and a final extension step performed at 72 °C for 10 min. After completion, the PCR reaction was purified with the GeneJET™ PCR Purification Kit (Fermentas UAB, Vilnius, Lithuania).

The purified fragments were enriched in microsatellite DNA containing sequences with the biotinylated probe (5′-Biotin-ATAGAATAT(CA)_16_), following a described procedure [11]. Basically, the Dynabeads^®^ M-270 Streptavidin coated magnetic beads (Dynal Invitrogen USA) were coupled with the biotinylated probe, then this complex was hybridized with the microsatellite DNA containing fragments which were fished out from the reaction mix with a magnet. The beads coupled with the microsatellite DNA containing fragments were further used as template in a PCR reaction aimed at increasing the number of fragments containing microsatellite DNA sequences. This PCR reaction was performed in a total volume of 50 μL, containing 8 μL of beads suspension, 10 mM Tris-HCl (pH8.8 at 25 °C), 50 mM KCl, 0.08% (v/v) Nonidet P40, 2.5 mM MgCl_2_, each dNTP at 0.2 mM, OligoA as a primer at 0.6 μM and 1 unit of Taq DNA polymerase (Fermentas UAB, Vilnius, Lithuania). The PCR conditions consisted of an initial denaturation at 95 °C for 3 min, followed by 5 cycles of 95 °C for 30 s, 60 °C for 30 s, 72 °C for 45 s, followed by 30 cycles of 92 °C for 30 s, 60 °C for 30 s, 72 °C for 55 s, followed by a final elongation step at 72 °C for 30 min.

The PCR products were purified using the Gene JET™ PCR Purification Kit (Fermentas UAB, Vilnius, Lithuania), ligated into the pJET1.2 vector using the CloneJET™ PCR Cloning Kit (Fermentas UAB, Vilnius, Lithuania), transformed into DH5α *Escherichia coli* competent cells, using the TransformAid™ Bacterial Transformation Kit (Fermentas UAB, Vilnius, Lithuania) and plated on LB agar plates containing 50 μg/μL ampicillin. The pJET1.2 vector used for cloning contains a lethal gene which is disrupted by the successful insertion of the cloned DNA fragment and, as a consequence, only the colonies containing DNA inserts grew on the agar plates.

The screening of these positive colonies for the identification of microsatellite DNA containing clones was performed by a PCR reaction with OligoA and a CA repeat oligo as primers. The OligoA primer yielded an amplicon the size of the insert, while the pair OligoA/Oligo CA yielded a second amplicon from the clones containing the CA repeat motif.

The PCR primers for each microsatellite locus were designed using Primer3 program [13]. One primer of each pair was modified by adding at the 5′ end a M13 tail to facilitate the genotyping on a LICOR 4300L system, using a labeled primer strategy, according to the producer’s specifications. The PCR genotyping reaction was performed in a 10 μL total volume containing about 50 ng of DNA template, 10 mM Tris-HCl (pH 8.8 at 25 °C), 50 mM KCl, 0.08% (v/v) Nonidet P40, 1.5 mM or 2.5 mM MgCl_2_, (see Table 1 for details for each locus) each dNTP at 0.1 mM, each primer at 0.1 μM, 0.02 μM of IRD700 or IRD800 labeled M13 primer and 0.5 units of Taq DNA polymerase (Fermentas UAB, Vilnius, Lithuania). The temperature profile of the PCR reaction consisted of an initial denaturation step at 95 ºC for 3 min followed by 30 cycles of denaturation at 95 °C for 30 s, annealing at a specific temperature for each locus (see Table 1 for details for each locus) for 30 s and extension at 72 °C for 45 s, followed by a final extension step at 72 °C for 5 min. After the PCR completion, 2 μL of formamide loading buffer was added to the samples followed by denaturation at 95 °C for 3 min, before loading them on a 6.5% polyacrylamide gel on a LICOR 4300 L genetic analyzer. The genotyping process was performed using Saga^GT^ v3.1 software package.

GenAlEx 6.4 was used to estimate the number of alleles per locus (N_A_), observed heteroygosity (H_obs_) and expected heterozygosity (H_exp_) [14]. Also, deviation from the Hardy-Weinberg equilibrium (HWE) was tested using the same software package. The presence of null alleles was tested using Micro-Checker (ver. 2.2.3) [15] while linkage disequilibrium test was carried out using Arlequin ver. 3.1 [16].

## Figures and Tables

**Table 1:** Primer sequences and characteristics of nine microsatellite loci successfully amplified in *Hypanis colorata*. T_a_, annealing temperature; [MgCl_2_], MgCl_2_ concentration in the PCR reaction; Size, range size of alleles.

Marker	Accession no.	Repeat motif	Primer sequence (5′-3′)	T_a_ (ºC)	[MgCl_2_]	Size (bp)
Hypo5	HQ696514	(TG)_31_	F: gtagtgggtttcggggaga	52	2.5 mM	268–394
			R: tctgcccaacacaaatggta			
Hypo10	HQ696515	(TG)_28_	F: tgcaacaaaacaggcaagaa	53	1.5 mM	166–280
			R: gcccgtatgaagcaaattgt			
Hypo11	HQ696516	(TG)_35_	F: ataaggtgtgcgtgcaagtg	50	2.5 mM	198–286
			R: cattctcacatgggttgctg			
Hypo12	HQ696517	(AACAG)_4_	F: gcggtgttggtcacacttatt	52	2.5 mM	166–266
			R: tctggtgtggtgtgaggtgt			
Hypo14	HQ696518	(AC)_3_AT(AC)_36_	F: caacaaagggcacaaacaag	53	1.5 mM	164–292
			R: catatccagagctggcttcc			
Hypo15	HQ696519	(AC)_1_AG(AC)_55_	F: ccccctgttgtaacgtgttt	52	2.5 mM	167–305
			R: cataccgccttttgtatgtcc			
Hypo2	HQ696520	(CA)_4_TA(CA)_4_CT(CA)_2_CGTA(CA)_24_	F: caaacacatccacgccaata	54	2.5 mM	200–264
			R: ttggacaatggatacacgtca			
Hypo9	HQ696521	(AC)_52_	F: gccattttgtgtcccagact	54	2.5 mM	180–292
			R: ggggcaatacatacctgagc			
Hypo13	HQ696522	(AC)_5_AT(AC)_3_AT(AC)_20_	F: gagaggggtcaggtcacaaa	54	2.5 mM	186–208
			R: gccggatgtatgtccaagtaa			

**Table 2:** Variation across 9 microsatellite loci in *Hypanis colorata*.

Marker	N	N_A_	H_obs_	H_exp_	HW-p
Hypo5	32	28	0.839	0.949	0.090
Hypo10	32	19	0.935	0.864	0.083
Hypo11	32	24	1.000	0.914	0.916
Hypo12	32	8	0.613	0.767	0.743
Hypo14	32	22	0.969	0.923	0.996
Hypo15	32	24	1.000	0.914	0.186
Hypo2	32	11	0.903	0.679	0.635
Hypo9	32	16	0.933	0.829	0.012
Hypo13	32	4	0.906	0.522	0.001*

N, sample size; N_A_, number of alleles; H_obs_, observed heterozygosity; H_exp_, expected heterozygosity; HW-p: probability value of chi square test for Hardy–Weinberg equilibrium;

* indicates significant departure (P < 0.05) from expected Hardy-Weinberg equilibrium conditions after correction for multiple tests (k = 9).

## References

[1] Popa OP Sarkany-Kiss A Kelemen SB Iorgu EI Murariu D Popa LO Contributions to the knowledge of the present Limnocardiidae fauna (Mollusca: Bivalvia) from Romania Trav. Mus. Nat. His. Nat. Gr Antipa 2009 52 7 15

[2] Munasypova-Motyash IA On the Recent Fauna of Subfamily Limnocardiinae (Bivalvia, Cardiidae) in North-Western Shore of Black Sea Vestnik zoologii 2006 40 41 48

[3] Panov VE Alexandrov B Arbaciauskas K Binimelis R Copp GH Grabowski M Lucy F Leuven RSEW Nehring S Paunovic M Semenchenko V Son MO Assessing the risks of aquatic species invasions via european inland waterways: From concepts to environmental indicators Integrated Environ. Assess. Manag 2009 5 110 126 10.1897/ieam_2008-034.1 19431296

[4] Grigorovich IA MacIsaac HJ Shadrin NV Mills EL Patterns and mechanisms of aquatic invertebrate introductions in the Ponto-Caspian region Can. J. Fish. Aquat. Sci 2002 59 1189 1208

[5] Allendorf FW Luikart G Conservation and the Genetics of Populations Blackwell Publishing Oxford, UK 2007

[6] Goldstein DB Schlotterer C Microsatellites Evolution and Applications Oxford University Press Oxford, UK 1999

[7] Li YC Korol AB Fahima T Beiles A Nevo E Microsatellites: Genomic distribution, putative functions, and mutational mechanisms: A review Mol. Ecol 2002 11 2453 2465 12453231 10.1046/j.1365-294x.2002.01643.x

[8] Selkoe KA Toonen RJ Microsatellites for ecologists: A practical guide to using and evaluating microsatellites markers Ecol. Lett 2006 9 615 629 16643306 10.1111/j.1461-0248.2006.00889.x

[9] Zhan A Bao ZM Hui MEA Inheritance pattern of EST_SSRs in self fertilizad larvae of the bay scallop Argopecten irradians Ann. Zool Fennici 2007 44 259 268

[10] Hedgecock D Li G Hubert S Bucklin K Ribes V Widespread null alleles and poor cross-species amplification of microsatellite DNA loci cloned from the Pacific oyster *Crassostrea gigas* J. Shellfish Res 2004 23 379 385

[11] Bloor PA Barker FS Watts PC Noyes HA Kemp SJ Microsatellite Libraries by Enrichment Available at: http://www.genomics.liv.ac.uk/animal/MICROSAT.PDF (accessed on 6 January 2011)

[12] Carleton KL Streelman JT Lee BY Garnhart N Kidd M Kocher TD Rapid isolation of CA microsatellites from the tilapia genome Animal Genetics 2002 33 140 144 12047227 10.1046/j.1365-2052.2002.00817.x

[13] Rozen S Skaletsky H Krawetz S Misener S Primer3 on the WWW for general users and for biologist programmers Bioinformatics Methods and Protocols Humana Press Totowa, NJ, USA 2000 365 386 10.1385/1-59259-192-2:365 10547847

[14] Peakall R Smouse PE GENALEX 6: Genetic analysis in Excel. Population genetic software for teaching and research Mol. Ecol Notes 2006 6 288 295 10.1093/bioinformatics/bts460 PMC3463245 22820204

[15] van Oosterhout C Hutchinson WF Wills DPM Shipley P Micro-checker: Software for identifying and correcting genotyping errors in microsatellite data Mol. Ecol Notes 2004 4 535 538

[16] Excoffier L Laval G Schneider S Arlequin ver. 3.0: An integrated software package for population genetics data analysis Evol Bioinformatics Online 2005 1 47 50 PMC2658868 19325852

